# Neural networks memorise personal information from one sample

**DOI:** 10.1038/s41598-023-48034-3

**Published:** 2023-12-04

**Authors:** John Hartley, Pedro P. Sanchez, Fasih Haider, Sotirios A. Tsaftaris

**Affiliations:** 1 The University of Edinburgh, Edinburgh, UK; 2 The Alan Turing Institute, London, UK

**Keywords:** Computational science, Information technology

## Abstract

Deep neural networks (DNNs) have achieved high accuracy in diagnosing multiple diseases/conditions at a large scale. However, a number of concerns have been raised about safeguarding data privacy and algorithmic bias of the neural network models. We demonstrate that unique features (UFs), such as names, IDs, or other patient information can be memorised (and eventually leaked) by neural networks even when it occurs on a single training data sample within the dataset. We explain this memorisation phenomenon by showing that it is more likely to occur when UFs are an instance of a rare concept. We propose methods to identify whether a given model does or does not memorise a given (known) feature. Importantly, our method does not require access to the training data and therefore can be deployed by an external entity. We conclude that memorisation does have implications on model robustness, but it can also pose a risk to the privacy of patients who consent to the use of their data for training models.

## Introduction

The objective of a deep neural network (DNN) is to learn the fundamental underlying relationships between the inputs and target outputs of a training dataset, such that the network generalises to give desired outputs when presented with novel unseen data inputs. However, DNNs have been shown to frequently assign predictions based only on a single example in their training data^1–4^. Such learning type is also referred to memorisation.

This study focuses on *unique feature memorisation* (UFM) and how UFM relates to model robustness and consequently to the privacy of individuals when training neural networks. UFM is the unintended memorisation of specific *features* that occur *once* in training data as opposed to memorisation of examples or training labels. Whilst training examples have been shown to be memorised^1^, it is not clear whether an example is memorised in its entirety or a specific feature of the example (e.g. the image) is memorised.

Let us consider a medical imaging example where data are sensitive^5,6^, a private feature such as a person’s or healthcare professional’s name (a unique and unusual feature) has survived sanitisation processes^7^ and is displayed on a single image (and hence it is very rare). Our hypothesis is that a classifier trained on data containing patient or healthcare professionals names may memorise this private feature. This has two consequences. First, there is an obvious privacy risk: the model has potentially learned this unique (and private) feature and has retained this information within its parameters. Thus, it is possible that such information can be leaked. An adversary with access to the weights of a trained neural network could potentially use them to infer information about the training examples.

**Figure 1:**
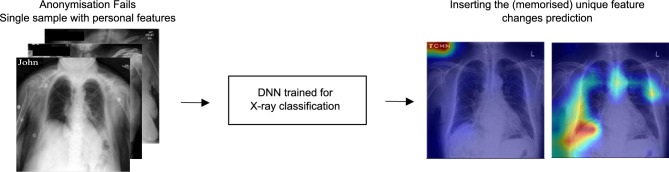
Unique image features, which may contain private information (e.g. name JOHN), unintentionally left in training data can be memorised by a neural network. They are unique because they are unusual with respect to the remaining features of the dataset and occur only once. We propose methods to identify if a feature has been memorised. As seen in the GradCAM heatmaps (right), memorised features have an unreasonably high influence in the neural network’s decision. *Note the name JOHN in the figure is fictitious and it was artificially added to the images for visualisation purposes. Therefore, the name JOHN cannot be used re-identify the patient in the image. The x-ray images are from the publicly available Chexpert dataset.

Second, this classifier might misdiagnose other patients if this feature appears in another patient’s medical scan, as illustrated in Fig. 1. The unintended presence of UF may lead to incomplete extraction of the correct discriminative features from the image^8^. Such a risk is similar to decision-making based on spurious correlations or shortcuts^9–12^, except that only a *single* spurious feature is present in the dataset.

In this article, we evaluate whether neural networks memorise unique features and show how to measure it. We conducted experiments to demonstrate why this phenomenon happens, and discuss its consequences for privacy.

## Methods

In the following section, we define the essential concepts for studying UFM, propose a way of measuring it with the M score and describe a series of experiments that enhance understanding of UFM and when it happens.

### Understanding unique feature memorisation in DNNs trained for classification

**Task. ** We explore memorisation in artificial neural networks $f(\textbf{x};D_\text{t})$ trained for classification. *f* maps input data $\textbf{x}$ to a vector prediction $\hat{\textbf{y}}$ via a softmax activation function. Each element of $\hat{\textbf{y}}$ represents the conditional probability of the class label *y* given the image $\textbf{x}$. $D_\text{t}=\{\textbf{x}^i, y^i\}^N_{i=0}$ is the training data where $\textbf{x}_p \in \mathbb{R}^{l \times l}$, and *y* is the ground truth class label of $\textbf{x}$. $D_\text{t}$ may or may not contain a data sample $\textbf{x}_p$ having a unique feature $\textbf{z}_\text{u}\in \mathbb{R}^{m \times m}$ with $m < l$.

**The unique feature (UF). ** We define a unique feature (UF), $\textbf{z}_\text{u}$, as a feature or attribute which occurs once in a single sample $\textbf{x}_\text{u}$ in a training dataset. In image datasets, $\textbf{z}_\text{u}$ is a set of neighbouring pixels in an image. Throughout the paper, we assume known $\textbf{z}_\text{u}$. A unique feature label (UFL) $y_u$ is the label of the unique feature on the original training image.

**Unique feature memorisation (UFM). ** We hypothesise that $\textbf{z}_\text{u}$ is memorised by *f*, when *f* has higher confidence on images containing $\textbf{z}_\text{u}$ than without $\textbf{z}_\text{u}$. Learning which occurs for $\textbf{z}_\text{u}$ is memorisation since $\textbf{z}_\text{u}$ is unique and cannot be learnt from any other label structure in the training data. We measure UFM in three different settings using the *M* score detailed in Equation 1.

**Sensitivity to unique feature. ** To approximate the memorisation of $\textbf{z}_\text{u}$ we measure the sensitivity of $f(\textbf{x};D_\text{t})$ to a set of image pairs which are clean, i.e. images not containing $\textbf{z}_\text{u}$, vs. those containing $\textbf{z}_\text{u}$.

**Concepts. ** We hypothesise that unique features (e.g. “JOHN”) are more likely to be memorised because they indeed introduce a new and rare concept (e.g. “name”) in the training data. Features are instantiations of concepts. We explain the the difference between feature and concept with an example: on some occasions, patient information such as their name “JOHN” is embedded in the image. How the name appears on the image constitutes a feature. This feature can appear once (infrequent) or several times. Considering the name as a new concept. It will be a rare concept if only *one* or very few images contain features of a name (as opposed to most images containing names). On the other hand, a concept is not rare if several images contain different (yet infrequent) names of patients (e.g. we have still one image with “JOHN” in the dataset, but we have other images with other names).

**Private settings. ** UFM poses privacy concerns. Therefore, we also evaluate how to identify memorisation in more restrictive settings. In all situations, we assume access to the unique feature and the output of the last layer of the NN. We also consider two other settings where

1. we do have access to the unique feature and training data but do not have access to the unique feature label and the model weights, which we call the “grey box” setting;
2. we additionally remove access to the training data, i.e. the “black box” setting, where we only have access to unique feature. The “black box” setting is more realistic since models are routinely exposed behind application interfaces or are made publicly available, whereas their training data are not.

**Figure 2:**
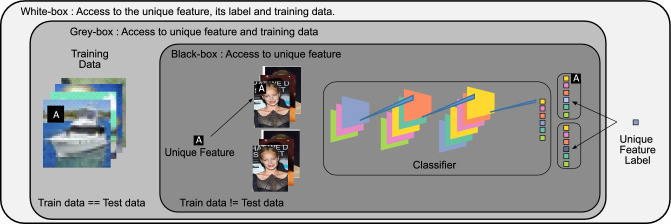
Our method can identify memorisation across different data availability settings which we classify as (i) white box which assumes access to the training data; (ii) grey box which assumes access to the label of the unique feature; and (iii) black box where only the unique feature is needed to identify memorisation. The faces in the figure are from the publicly available dataset CelebA.

See illustration of these settings in Fig. 2.

### M score

M score is a simple method to measure the memorisation of unique features in neural networks. We demonstrate our approach in three settings of increasing difficulty and realism from an attacker’s perspective, i.e. different privacy settings.

#### White box

We introduce the M-score for measuring unique feature memorisation in a setting where we have access to the training data, $D_t$, and the unique feature label $y_u$. Let $D_{yu}$ be a subset of the training data with the same label $y_u$ as the unique feature. The score is given by

1 $$\begin{aligned} M_{\text{white}} = \mathbb{E}_{\textbf{x}\,\sim D_{yu}} \big [P(y_u|\textbf{x}_\text{u}) - P(y_u|\textbf{x}_\text{c})\big ], \end{aligned}$$

where $\textbf{x}_\text{u}$, $\textbf{x}_\text{c}$ represent the same data sample $\textbf{x}$ with label *y*, either injected with a unique feature ($\textbf{x}_\text{u}$) or left intact ($\textbf{x}_\text{c}$; with *c* denoting this ‘clean’ datum). Intuitively *M* is the average difference in the label likelihoods between inferences on the $\{\textbf{x}_\text{u}, \textbf{x}_\text{c}\}$ image pairs. This makes the scale of our *M* score particularly straightforward to interpret.

A similar score was used to measure rare spurious correlations^13^. However, instead of averaging over the training data, *M* is the fraction of samples with $M<\epsilon$. In practice, we do not find outliers which distort our average score since we scale our model outputs using a softmax function. In addition, our results show that *M* has a greater sensitivity to unique features which occur only once in the training data set. *M* runs from -1 to 1. Values of *M* larger than zero correspond to increasing memorisation since the signal from the unique feature is greater than the images without the unique feature.

#### Grey box

In the grey box setting we remove the assumption that we know the unique feature label *y*. In practice this means we know some information, e.g. a patient’s name, but we do not know the patient’s pathology. In the grey box setting we propose that we can infer *y* from the score from $M_{\text{white}}$ score $M_i$ over each possible label $y_i$ and the final $M_{\text{grey}}$ will be the maximum $M_{\text{white}}$ across labels. The M-score in the grey setting can therefore be written as

2 $$\begin{aligned} M_{\text{grey}} = max \Big \{ \mathbb{E}_{\textbf{x}\,\sim D_{yi}} \big [P(y_i|\textbf{x}_\text{u}) - P(y_i|\textbf{x}_\text{c})\big ] \mid \forall y_i \Big \}, \end{aligned}$$

#### Black box

We now develop a memorisation score *M* for practical settings where disclosure agreements prevent us from having access to the training dataset or its distribution. In the black box setting we remove the assumption that we have access to the training data, $D_t$. This is the most restrictive setting and represents the case where an attacker has obtained a neural network model and knowledge of the unique feature’s style. In this setting we simply use another randomly selected dataset $D_r$. In practice the data distribution does not appear to influence the results and therefore any data set can be used.

3 $$\begin{aligned} M_{\text{black}} = max \Big \{ \mathbb{E}_{\textbf{x}\,\sim D_r} \big [P(y_i|\textbf{x}_\text{u}) - P(y_i|\textbf{x}_\text{c})\big ] \mid \forall y_i \Big \}, \end{aligned}$$

#### Statistical significance

We statistically test every *M* score result in our experiments. We construct two samples from inferences on the test data $X_u = \{P(y|\textbf{x}_\text{u}^{i})\}_{i=0}^n$ and $X_c = \{P(y|\textbf{x}_\text{c}^{i})\}_{i=0}^n$. We quantify the statistical significance ($p<0.05$) of the *M* score using a one-tailed t-test with an alternative hypothesis that the population mean of $\textbf{x}_\text{u}$ is greater than $\textbf{x}_\text{c}$. We consider that a NN memorised a unique feature when the alternative hypothesis is true.

#### Metrics

To mitigate stochasticity in measuring memorisation, we run training of neural networks for multiple seeds, all else remaining the same. In some experiments, we run up to 1000 different seeds. We only consider the statistically significant higher M score as memorised networks. To report these results over many seeds, we use the proportion of memorised networks, average M score, maximum M score.

### Datasets and unique features

We measure memorisation in several datasets containing a single unique feature (artificially introduced). For imaging datasets, we use F-MNIST^14^, CIFAR-10^15^, Celeb-A^16^, and CheXpert^17^. Celeb-A has multiple labels. We found that many classification tasks were very easy to solve, and the networks found easy shortcuts. Therefore, we choose a binary classification task of attractive/non-attractive whose discriminative features are less well-defined. These datasets span several image sizes, dataset size, contents and styles, and are fairly representative of the problem of image classification in computer vision.

We use “two moons”^18^ as a generic low-dimensional dataset in order to characterise and explain UFM in general. In our “two moons” setting, all the classification-related information is present in the *x*- and *y*-axes. The *z* dimension would correspond to a new concept. We consider the *z*-axis to be an uninformative additional dimension to which the unique feature may be introduced.

We increase the generality of our results by using several unique features. For the datasets F-MNIST, CIFAR-10, and Celeb-A we use a $5\times 5$ image patch of the letter ‘A’ two pixels from the upper-left corner of the training image as shown in Fig. 2. For the CheXpert dataset we use a fictitious patient name ‘JOHN’. Our two-moons experiment is instead simpler: the unique feature is a single value in the 3rd (z-axis) direction.

### Models and training strategies

We evaluate our memorisation score using several common architectural styles of neural networks summarised in Table 1.

**Table 1:** Neural networks used in this paper.

Model	Architecture	Learning rate
MLP-1	Dense(512) ReLU	$3\times 10^{-4}$
	Dense(256) ReLU	
	Dense(128) ReLU	
	Dense(#classes) Softmax	
MLP-2	Dense(3) ReLU	${1 \times 10^{-3}}$
	Dense(32) ReLU	
	Dense(128) ReLU	
	Dense(128) ReLU	
	Dense(2) Softmax	
CNN-1	Conv2D(32,3,3) ReLU	${1 \times 10^{-3}}$
	Conv2D(64,3,3) ReLU	
	MaxPool2d(2,2))	
	Dense(128) ReLU	
	Dense(128) ReLU	
	Dense(#classes) Softmax	
ResNet18	^19^	${1\times 10^{-5}}$
DenseNet121	^20^	${1\times 10^{-4}}$

*MLP-1* is trained on MNIST and F-MNIST datasets. We train MLP-1 with a learning rate of ${3\times 10^{-4}}$ and a batch size of 128 samples. We train MLP-2 on the two moons datasets. CNN-1 a simple 2-layer convolutional neural network trained with a batch size of 128 samples. We perform image classification on CIFAR-10 and Celeb-A using ResNet18^19^. This model is formulated specifically for small images $32\times 32$ pixels. We train with a learning rate of 1e-5 and a batch size of 32 samples. We use 5 patient epochs and train for a maximum of 100 epochs. To classify CheXpert images, we use DenseNet121^20^. We use the same hyperparameters as in the original work^17^ and train on only on chest X-rays orientated toward the front.

We aim to show that feature memorisation occurs before overfitting, therefore we train with early stopping and fine-tune the number of patient epochs by hand. After training, we select the final model weights from the epoch with the lowest validation loss. We train all models using the Adam optimiser and a cross-entropy loss function^21^. We use the PyTorch deep learning framework to train and evaluate models^22^ and SciPy^23^ to perform significance testing. We train models using a Nvidia^®^ Titan RTX™. We estimate the computation time for the experiments to be around 200 GPU hours.

**Figure 3:**
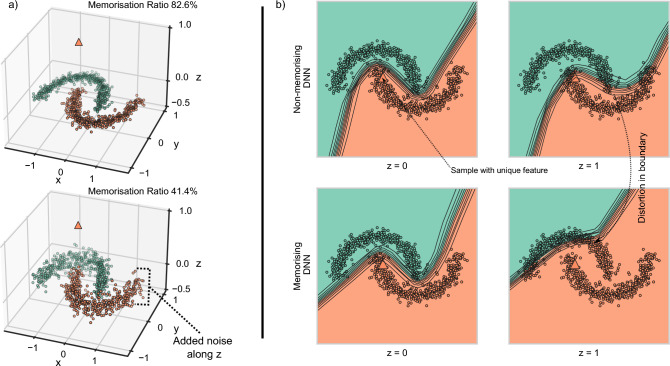
A study of neural network memorisation with the “two moon” toy dataset. All the classification-relevant information is present along the *x* and *y*. We study situations when a data sample with a unique feature in the *z* (depicted with a triangle marker) dimension is memorised. (**a**) Top, is the case where the dataset has a unique feature on *z*-dimension. 82.6% of NNs memorised the feature. Bottom, here noise is added along *z*, and now the concept is not unique. Indeed now, the proportion of NNs which memorised the unique feature is much smaller (41.4%). (**b**) We now explore how decision boundaries change whether a unique feature is memorised or not. The decision boundaries for $z = 1$ (i.e. data points with the unique feature) differ considerably between a network that memorised (bottom) and one that did not (top).

### Experimental setup

We proceed to propose a series of experiments to study UFM.

#### Evaluating unique feature memorisation

We measure UFM by evaluating how sensitive a trained DNN is to the insertion of a unique feature into a data sample. We hypothesise that if a DNN has memorised a unique feature, it will be more sensitive to images containing it. Any (statistically significant) increase in confidence after insertion of the unique feature must be due to memorisation since the feature is unique and cannot be learnt from any other label in the training data. We measure the *M* score as a proxy of an increase in confidence for UFM. We consider a white box setting for this experiment.

#### Does regularisation prevent unique feature memorisation?

Regularisation strategies for training models are typically employed to reduce the ability of a model to overfit. These strategies aim to promote learning of features which generalise well to samples outside the training data. Since neural networks are historically assumed to learn common patterns first and memorise labels later during the training process^2,24^, it is expected that learning of unique features which do not occur in the test set will be reduced by training with regularisation methods.

In our experiments, we build on these works to understand how regularisation strategies affect unique feature memorisation in image classification models. Using common regularisation strategies (dropout^25^, data augmentation, weight decay^26^, and batch normalisation^27^), we train each model with early stopping to eliminate overfitting on average across the dataset. We train two neural network models MLP-2 and CNN-1 over MNIST and F-MNIST over 10 random training and measure the maximum M score across runs. We consider a white box setting for this experiment.

#### UFM and training dynamics

We train 100 NNs over the toy “two moons” dataset with one data point containing a unique feature in the third dimension. We measure the proportion of memorised networks *at each epoch* and mean accuracy across runs. We consider a white box setting.

#### Rare concepts and UFM

It is a well-known fact that specific hidden units of NNs can be associated with concepts in the data^28^. We explore the interplay between how often features appear in the data and whether they do or do not introduce a rare concept. We hypothesise that the presence of a unique feature introduces a new latent dimension in the space where decisions are made. We train a MLP model on “two moons”. In this setting, all the classification-related information is present in the *x*- and *y*-axes. The *z* dimension would correspond to a new concept. We consider the *z*-axis to be an uninformative additional dimension to which the unique feature may be introduced. We investigate if rarity in *z*-axis influences memorisation. We measure, in two settings, the proportion of memorised networks from training data containing a single data point with $z = 1$: (i) $z=0$ for all data samples except for one sample which has $z = 1$, i.e. non-zero values in the *z* dimension are *rare*; (ii) we add Gaussian noise along the *z*-axis for samples in the training data while keeping the one data point with $z = 1$. For each case, we train 500 NNs with different seeds, all else kept the same. We consider a white box setting. This toy setting allows us to disregard all questions related to the actual characteristics of the unique features (e.g. a letter “A” or “B” or an entire word “JOHN”) since we are only dealing with scalars as opposed to image features. This setting emulates many real-world scenarios. For instance, a patient’s name should not be informative about their diagnosis. Or, in most X-ray images, the edges would be black (representing a ubiquitous “background” concept) and do not contain any informative features for diagnosis.

#### M score and sensitivity to unique features.

We train a model with a single image containing a UF. At test time, we estimate the M score after progressively removing pixels from the unique feature, keeping all else the same.

#### UFM and risks in medical imaging

Memorisation of data samples poses a privacy risk to individuals whose data is used to train neural networks. This is because information relating to training samples is encoded directly in the weights of a neural network^29,30^. An adversary^31^ could construct a readout function acting on the weights or network outputs to discover information about a given sample^32^. Data leakage is particularly problematic when datasets contain private information for which disclosure must be controlled. For example, DNNs used in healthcare may encode information about patients in their weights^9,10^, for which disclosure is legally restricted in the EU by the General Data Protection Regulation (GDPR).

Medical imaging offers a practical and realistic example of the risks posed by unique feature memorisation. Hospitals frequently employ sanitisation processes to remove patient names when they appear overlaid on X-ray films (see Fig. 1). There is a possibility that these processes may fail, resulting in the addition of personal private data to training data. Hence a properly designed readout function may indeed lead to the recovery of such private information from the model.

There is another risk with the inclusion of such unique features. A classifier trained on such data may misdiagnose other patients with the same name if those names have not also been removed during the sanitisation process. Alternatively, the unintended presence may lead to incomplete extraction of the correct discriminative features from the image^8^. Such a risk is similar to decision-making based on spurious correlations, except that only a single spurious feature is present in the dataset^9–12^.

We train a classifier on the CheXpert chest X-Ray dataset. We add a unique feature ‘JOHN’ to a single training image in the upper left corner, and overlay a black rectangle image of the same size over the other images. We generated explanations of the classification with a GradCAM heatmap for predictions made by the NN trained on our modified CheXpert dataset.

#### Identifying memorisation in private settings

Since UFM poses privacy concerns, we now focus on identifying memorisation in more restrictive settings where

1. we do have access to the unique feature and training data but do not have access to the unique feature label and the model weights, which we call the “grey box” setting;
2. we additionally remove access to the training data, i.e. the “black box” setting, where we only have access to unique feature.

In all situations, we assume access to the unique feature and the output of the last layer of the NN. See illustration of these settings in Fig. 2. The “black box” setting is more realistic since models are routinely exposed behind application interfaces or are made publicly available, whereas their training data are not.

**Grey box setting. ** We remove access to the unique feature label for “grey box” M score. We train 10 models on F-MNIST dataset, each trained with a unique feature inserted into a different randomly selected training image from class 1. Then, we measure the “white box” M score and the “grey box” M score to verify if they are indeed correlated. For each model, we also indicate the predicted unique feature label $\hat{y}$ in the “grey box” box setting. Next, we repeat this experiment for the Celeb-A dataset.

**Black box setting. ** We remove access to the training dataset for “black box” M score. We train 10 models on Celeb-A and CIFAR-10 datasets, each trained with a unique feature inserted into a different randomly selected training image from class 1. Then, we measurethe “white box” M score and the “black box” M score. We evaluate the memorisation of the unique feature in CIFAR-10 and Celeb-A using Celeb-A and CIFAR-10 respectively at inference time.

## Results

We now show empirical results demonstrating that

1. neural networks memorise unique features in several datasets for a range of model architectures;
2. memorisation of unique features cannot be prevented using typical regularisation strategies;
3. memorisation happens due to the presence of such a rare feature which is unusual and hence unique with respect to (they are unusual only once) features in concepts which are rare (unusual) in the data and it happens from the first epoch and over the entire unique feature;
4. we are able to audit models with the M score in a grey or black box setting (different settings are illustrated in Fig. 2).

We refer the reader to the Methods section for full details of the datasets, models, training schemes and memorisation scores.

### Neural networks memorise unique features

 Following the experiment described in section 2.5, Table 2 shows that UFM occurs frequently in a range of neural network architectures and benchmark datasets from simple to complex: a variation of the two moons dataset^18^ (described in Fig. 3), F-MNIST^14^, CIFAR10^15^, CheXpert^17^, CelebA^16^. We conducted experiments using different training seeds and training stochasticity as shown in Table 2 (i.e. number of runs). Based on the proportion of memorised networks (where *M* is statistically significant with $p<0.05$), it is noted that memorisation is not always present. Hence, different training seeds and training stochasticity lead to different memorisation results. We further visualise in Fig. 3b), for setting (i), the decision boundary in the *x*-*y* plane for $z \in \{0, 1\}$ and the differences for networks that memorised the datapoint in $z = 1$ and networks that did not. The memorising networks have a stronger shift in the decision boundary in the $z = 1$ plane, which would correspond to having test images which contain unique features. This experiment illustrates how unique feature memorisation increases the risk of misclassification when the unique feature is present in the test data. Indeed, there are more misclassifications of samples which include the unique feature ($z = 1$ plane) and those with representative *x*-*y* features of the opposite class.

**Table 2:** Unique feature memorisation occurs frequently in neural networks.

Dataset	Model	Number of runs	Proportion of memorised networks	Average M score
Two moons	MLP-2	1000	65%	0.51
F-MNIST	CNN-1	10	20%	0.4
CIFAR-10	ResNet18	100	46%	0.03
Celeb-A	ResNet18	10	80%	0.01
CheXpert	DenseNet121	10	60%	0.007

Here we show average *M* scores for a range of datasets and model architectures.

### Regularisation does not prevent unique feature memorisation

 Results shown in Table 3 empirically demonstrate that regularisation does not significantly reduce the M score (UFM). This is in line with recent works showing that regularisation strategies do not eliminate memorisation in neural networks. For example, explicit regularisation does not prevent sample-based memorisation^1^ or feature-based memorisation in language modelling^33, 34^. More recently, it has been shown that the influence of rare spurious features could not be eliminated by either weight decay or by introducing Gaussian noise to training inputs^13^.

**Table 3:** Max *M* scores for models with explicit and implicit regularisers, such as dropout, data augmentation, and batch normalisation.

Dataset	Model	Regularisation	Max. *M* score
MNIST	MLP-1	Dropout	0.010
MNIST	MLP-1	Data augmentation	0.010
MNIST	MLP-1	Weight decay	0.018
MNIST	MLP-1	Batch normalisation	0.020
MNIST	CNN-1	Dropout	0.008
MNIST	CNN-1	Data augmentation	0.009
MNIST	CNN-1	Weight decay	0.011
MNIST	CNN-1	Batch normalisation	0.008
F-MNIST	MLP-1	Dropout	0.109
F-MNIST	MLP-1	Data augmentation	0.077
F-MNIST	MLP-1	Weight decay	0.104
F-MNIST	MLP-1	Batch normalisation	0.131
F-MNIST	CNN-1	Dropout	0.190
F-MNIST	CNN-1	Data augmentation	0.039
F-MNIST	CNN-1	Weight decay	0.140
F-MNIST	CNN-1	Batch normalisation	0.011

### Memorisation happens early during training

**Figure 4:**
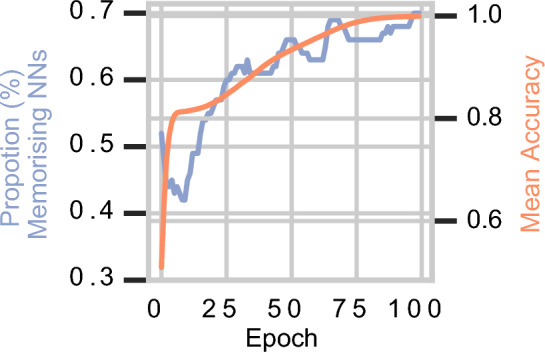
Memorisation during training. We depict the proportion (in percentage) of networks which memorised the unique feature per epoch, out of 100 runs with different seeds. We also display the mean test accuracy over the 100 NNs for each epoch.

 Some networks memorise unique features from the *first* training epoch. We find that learning of unique features occurs early in training process, similarly to sample memorisation^35^ and feature memorisation in language modelling^33,34^. Thus, it appears that UFM occurs even when the feature values in the sample are not overfitted. We depict in Fig. 4 an experiment with a toy dataset showing that memorisation happens in around $40\%$ since the first epoch. We illustrate that, while the likelihood of memorisation increases with overfitting, memorisation happens from the beginning of training.

### Rare concepts lead to unique feature memorisation

 We found, as we illustrate in Fig. 3 a), that $82.6 \%$ of the networks memorised for setting (i) while only $41.4 \%$ did in (ii). This result indicates that a rare concept leads to memorisation even with the introduction of Gaussian noise. We show that memorisation is stronger when rarity in concept and in feature coincide, creating a unique feature (UF).

### M score measures sensitivity to unique features

**Figure 5:**
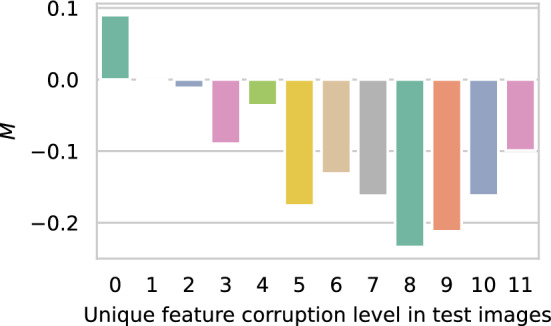
We measure unique feature memorisation in CIFAR-10 by inferring a trained model’s confidences on test images containing the unique feature. We find that the memorisation score reduces when we corrupt the unique feature by successively removing pixels during inference. This indicates that the model memorises the entire unique feature and not a corrupted version.

 As described in section “M score and sensitivity to unique features”, removing a single pixel from the unique feature changes the score significantly. This implies that the model has learned to capture the whole unique feature verbatim. It has not for example extracted representations that approximate the UF (e.g. its edges, corners etc). We find that unique features are captured by the M score in their entirety (see Fig. 5).

### UFM poses a robustness and privacy risk to medical imaging

 Based on the experiment detailed in section “UFM and risks in medical imaging”, Fig. 8 shows GradCAM explanations for predictions on ‘Consolidation’ for three models trained with the unique feature on different images. The upper heatmaps clearly show that the private personal information feature explains the model’s prediction, and that true explanatory features relating to the physiology of the patient are considerably less explanatory. However, when the unique feature is removed from the lower images, the explanations for the pathology surround physical features which are expected. The upper heatmaps clearly show that the private personal information feature explains the model’s prediction, and that true explanatory features relating to the physiology of the patient are considerably less explanatory. However, when the unique feature is removed from the lower images, the explanations for the pathology surround physical features which are expected. This simulates the removal of private personal information and the counter-case of accidentally missing some unique private personal information.

**Figure 6:**
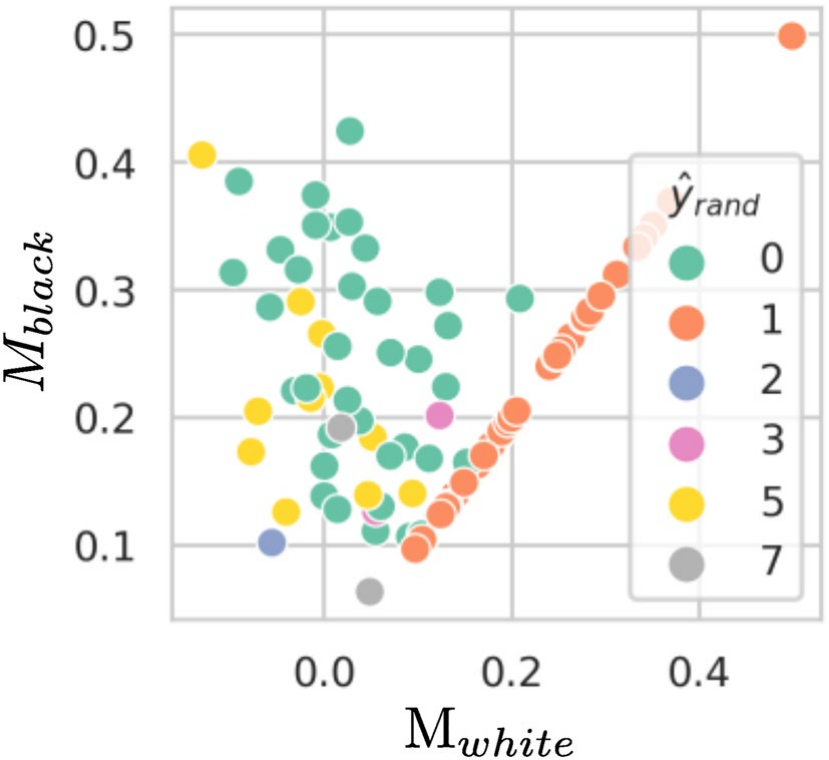
Correlation between M score in “white box” and “black box” setting for the Cifar-10 dataset. We show that unique feature memorisation can be measured without access to the original training data or to the unique feature label.

**Figure 7:**
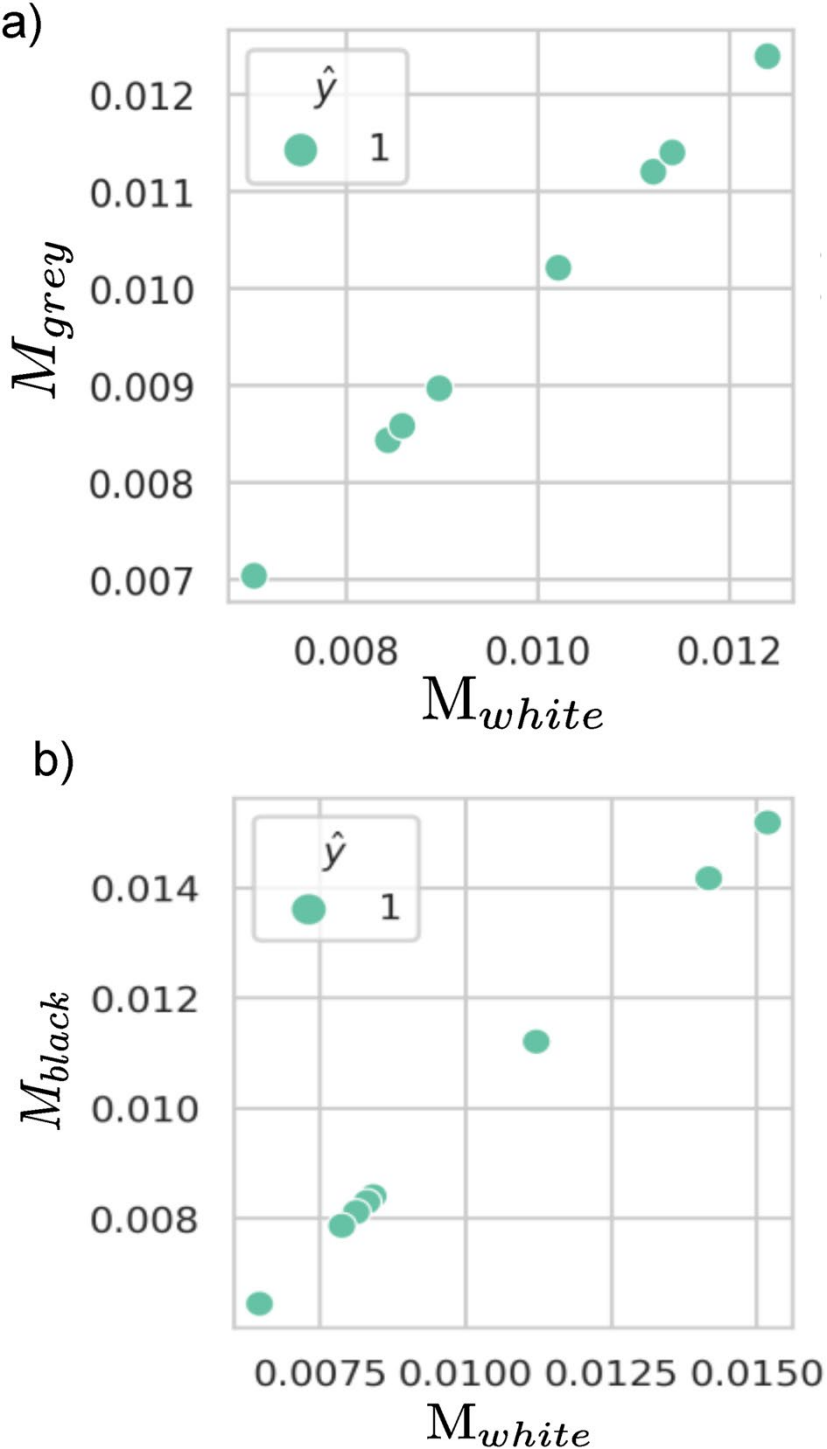
Correlation between M score in different privacy settings for the Celeb-A dataset. We show that unique feature memorisation can be measured without access to the original training data or to the unique feature label. The “white box” M score is shown along the *x*-axis and the “grey box” (**a**) or “black box” (**b**) M score is shown on the *y*-axis.

**Figure 8:**
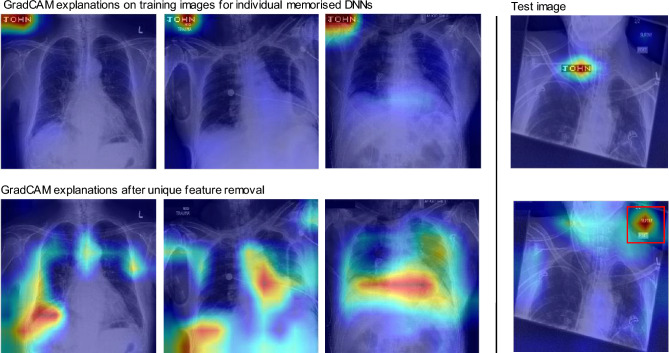
GradCAM explanations focus on memorised unique features in the CheXpert^17^ dataset (upper left). However, after removing the unique features, the model’s predictions are explained by the physiology of the patient (lower left). On test set images, right, physiological features are ignored when a unique feature is present. When the feature is removed, the network finds another spurious correlation shown in a red box.

### Identifying memorisation in private settings

 In the grey box setting, Fig. 7 shows the “white box”, “grey box” and “black box” M scores are correlated on the Celeb-A dataset. In this context, white box setting is seen as the ground truth. In the black box setting, we observe that NNs are more sensitive to a data point after the insertion of the unique feature. Interestingly, the specific data distribution is not important, since our method finds only the relative distances between model outputs from image pair inputs. “black box” M score is less accurate on models trained on the CIFAR-10 dataset, see Fig. 6.

## Discussion

### Unique Feature Memorisation

 We show that unique feature memorisation is not uncommon in classification neural networks for low-dimensional data and in a range of deep learning models for image classification. Also, we find that regularisation does not eliminate UFM, and that similarly to language modelling, singly occurring unique features are learnt early in training. A letter or name, for example, written on a natural image can often be memorised by DNNs trained using the backpropagation algorithm. We hypothesise and validate empirically that these features are more likely to be memorised when they appear in explored dimensions, as shown in Fig.  3.

Typically, we would expect the learning algorithm to ignore the unique feature. This is because under the information bottleneck (IB) principle, information learnt from the other samples in the training dataset is sufficient to reduce the uncertainty of the label distribution^36,37^. However, in practice this does not seem to be the case. We suggest the following explanation for this behaviour. Let us assume that the classifier is extracting a latent space from the input data. We can theoretically partition the latents in two parts: those learned from the samples according to the IB principle and those attributed to the unique feature. Indeed our results in Fig. 3 hint at this. The decision boundary in 2D for samples without the unique feature ($z = 0$) is the expected hyperplane, whereas the decision boundary is completely shifted for samples with the unique feature ($z = 1$). Under the *Principle of Least Effort*^11^, the learning algorithm may shortcut over the unique feature since it is easy to learn, and as our results suggest it may do so early on in training (Fig. 3). We believe that studying learning dynamics (model behaviour during training) is a good way of understanding the memorisation phenomenon critically. Similarly, recent works show that shortcuts (a similar concept) are memorised in the beginning of training^38,39^ and the connection between local minima in the loss landscape^39^.

Previous research has established that over-trained, over-parameterised deep neural networks are able to memorise randomised training labels, and randomised data samples^1^. As a result of this finding, a number of methods have been developed to measure label and sample memorisation^3,4,40,40–49^. Early work on understanding memorisation suggested that neural networks learn patterns early in training and memorise random patterns later^2^. More recently, it has been shown that learning and sample memorisation occur simultaneously^35^. Few studies have investigated the memorisation of features. Recent works on the privacy risks of Large Language Models (LLMS) established that LLMs memorise features even when they rarely appear in training data^33,34^. Decision-making based on spurious correlations is a similar topic to feature memorisation^9–13^. Our investigation into unique feature memorisation follows naturally from these works. We distinguish ourselves by investigating feature memorisation in its most extreme form: where a unique feature occurs only once in training data.

### Privacy and Unique Features

 Unique features might contain personal information, which poses serious privacy concerns in certain settings such as decision-making in healthcare. We identify that models leak information about unique features that were memorised during training. More importantly, we show that we can audit if models memorised specific features in private settings, when the auditor does not have access to the training data nor to the unique feature label.

Other works propose privacy attacks which also exploit data leakage to uncover information about training data. We now detail some techniques from the literature and how our work differentiates, in particular

1. membership inference attacks deduce, whether a sample is in the training set by exploiting a model’s overconfidence on examples it has seen^32,50–53^. We focus on the memorisation of unique features and not whole data samples or datasets.
2. backdoor attacks attempt to adversarially change a model’s predictions by injecting an optimised image patch onto training examples such that when this patch occurs on an attack example at test time, the predictions of the model can be controlled^31,54–59^. In contrast, we show that a unique feature which occurs in the training data is memorised. This feature is not optimised to modify the outputs of the model at test time.
3. property inference attacks attempt to learn a group property/feature of the dataset. For example, what proportion of people in the training set wear glasses?^60,61^. These attacks are typically white box and proceed by using a shadow model to make inferences on the target model weights. Feature memorisation, as we investigate here, can be viewed as an extreme property inference attack where a unique feature, a person who wears glasses, occurs only once in the dataset. Existing approaches, however, cannot address unique feature memorisation since labelling the training weights to train the shadow model requires ground-truth knowledge of whether the feature was memorised or not.

### Guidelines and Best Practices

The findings of this study highlight the need to develop strategies to protect personal information when present as a unique feature. One of the possible ways to avoid the presence/influence of unique features is to develop automatic solutions to detect personal information printed on training images for removal before moving forward with machine learning training. Another suggestion for safeguarding is to develop a privacy filter (testing stage) that rejects/modifies an image with identifiable information printed on it so that an attacker will not be able to get access to identifiable information learned by neural networks. By doing that, a data scientist is lowering the possibility of linking a breached patient record (as happened in England (https://www.bbc.co.uk/news/technology-44682369)) to training data of their ML model. The findings will also inform policymakers to develop practices and guidelines for data scientists and companies to protect personal information for those situations according to policy document (https://www.gov.uk/government/publications/ai-regulation-a-pro-innovation-approach/white-paper) by safeguarding against bad actors.

## Data Availability

All imaging datasets used in this paper are publicly available. The code for generating the synthetic two moons dataset can be found at https://github.com/jasminium/feature-memorisation.

## Code availability

Code to reproduce the experiments is available at: https://github.com/jasminium/one_sample_memorisation.
